# Transcriptome analyses of seed development in grape hybrids reveals a possible mechanism influencing seed size

**DOI:** 10.1186/s12864-016-3193-1

**Published:** 2016-11-09

**Authors:** Li Wang, Xiaoyan Hu, Chen Jiao, Zhi Li, Zhangjun Fei, Xiaoxiao Yan, Chonghuai Liu, Yuejin Wang, Xiping Wang

**Affiliations:** 1 State Key Laboratory of Crop Stress Biology in Arid Areas, College of Horticulture, Northwest A&F University, Yangling, Shaanxi 712100 China; 2 Key Laboratory of Horticultural Plant Biology and Germplasm Innovation in Northwest China, Ministry of Agriculture, Northwest A&F University, Yangling, Shaanxi 712100 China; 3 Boyce Thompson Institute for Plant Research, Cornell University, Ithaca, NY 14853 USA; 4 Zhengzhou Fruit Research Institute, Chinese Academy of Agricultural Sciences, Zhengzhou, China

**Keywords:** Grape, Hormone, Mechanism, Seed size, Transcriptome

## Abstract

**Background:**

Seedlessness in grape (*Vitis vinifera*) is of considerable commercial importance for both the table grape and processing industries. Studies to date of grape seed development have been made certain progress, but many key genes have yet to be identified and characterized.

**Results:**

In this study we analyzed the seed transcriptomes of progeny derived from the *V. vinifera* seeded maternal parent ‘Red Globe’ and the seedless paternal parent ‘Centennial seedless’ to identify genes associated with seedlessness. A total of 6,607 differentially expressed genes (DEGs) were identified and examined from multiple perspectives, including expression patterns, Gene Ontology (GO) annotations, pathway enrichment, inferred hormone influence and epigenetic regulation. The expression data of hormone-related genes and hormone level measurement reveals the differences during seed development between seedless and seeded progeny. Based on both our results and previous studies of *A. thaliana* seed development, we generated network maps of grape seed-related DEGs, with particular reference to hormone balance, seed coat and endosperm development, and seed identity complexes.

**Conclusion:**

In summary, the major differences identified during seed development of seedless and seeded progeny were associated with hormone and epigenetic regulation, the development of the seed coat and endosperm, and the formation of seed identity complexes. Overall the data provides insights into the possible molecular mechanism controlling grape seed size, which is of great importance for both basic research and future translation applications in the grape industry.

**Electronic supplementary material:**

The online version of this article (doi:10.1186/s12864-016-3193-1) contains supplementary material, which is available to authorized users.

## Background

Seed development in angiosperms is initiated by double fertilization, in which two sperm cells separately fuse with the egg and the central cell of the female gametophyte, leading to the formation of a diploid embryo and a triploid endosperm [1]. This process occurs inside the ovule that, following fertilization, develops into a seed. The seed coat, which is derived from ovular tissue, is a multifunctional structure that plays an important role in protecting the embryo, and regulating seed germination and embryo nutrition during seed development [2]. In the seed interior, the endosperm mediates the transfer of nutrients from the maternal parent to the embryo, which after cellularization begins to absorb the endosperm, resulting in its disappearance and the development of the cotyledons, which function as storage organs [3].

Development of ovule, precursor of seed, has been well studied in the model plant *Arabidopsis thaliana*, where it has been associated with a coordinated series of physiological and biochemical events [4]. Genetic studies have identified many of the genes that participate in *A. thaliana* ovule development, such as *SHOOT MERISTEMLESS* (*STM*), *CUP-SHAPED COTYLEDONS1* (*CUC1*) and *CUC2*, which are involved in meristematic cell maintenance [5–8]. There is also evidence that *AINTEGUMENTA* (*ANT*) is required specifically to promote the growth of the placenta to allow ovule primordium formation [9], and mutations in *BELL1 (BEL1)*, which is required for integument morphogenesis, affect ovule identity [10, 11]. Furthermore, the MADS-box genes *AGAMOUS* (*AG*), *SEPALLATA* (*SEP1*, *SEP2* and *SEP3*), *SHATTERPROOF* (*SHP1* and *SHP2*) and *SEEDSTICK* (*STK*, also known as *AGL11*) share a common function in promoting ovule and seed identity [12–15].

Genetic and molecular analyses have also shown that *A. thaliana* seed size is affected by both the seed coat and endosperm development [16–18]. For instance, an adaxial–abaxial polarity mechanism is required for formation of the integument, which later differentiates to form the seed coat [19, 20], and several genes have been identified that contribute to establishing this polarity. As an example, *INNER NO OUTER* (*INO*), a member of the YABBY family, is required for outer integument formation, as are the *KANADI* genes, *KAN1* and *KAN2* [21, 22]. *ABERRANT TESTA SHAPE*/*KANADI4* (*ATS*/*KAN4*) is known to be expressed in the abaxial domain of the inner integument and *NOZZLE*/*SPOROCYTELESS* (*NZZ*/*SPL*), *Knotted1-like Homeobox* (*KNOX*) and *PHABULOSA* (*PHB*) have been shown to be important in coordinating inner and outer integument formation [23–26], which further developed into seed coat. On the other side, the molecular characterization of *HAIKU2* (*IKU2*) and *MINISEED3* (*MINI3*) in small seeded mutants provided direct molecular evidence that the regulator of seed size acts solely through the control of endosperm proliferation [27]. In addition, imprinting provides a key mechanism in the modulation of endosperm development, and involves a large group of polycomb (PcG) proteins, including FERTILIZATION INDEPENDENT SEED 2 (FIS2), FERTILIZATION-INDEPENDENT ENDOSPERM (FIE/ FIS3), MEDEA (MEA/FIS1), MULTICOPY SUPRESSOR OF IRA (MSI1), and SWINGER (SWN), which form polycomb repressive complexes that suppress gene expression through histone and DNA methylation [28]. In summary, genes involved in establishing polarity, meristem maintenance, floral organ determination, ovule and seed identity, structure specification, and epigenetic regulation have all been shown to be important for seed development.

Seed development has also been studied in grapevine (*Vitis vinifera* L.), an important fruit crop in many parts of the world, and seedless grapes valued as both table grapes and for raisin production. Grape seedlessness is caused by either parthenocarpy or stenospermocarpy. In our study, all the seedless materials used were stenospermocarpy, which means both pollination and fertilization occur but both the seed coat and endosperm cease their normal development at early stages, leaving undeveloped seeds or seed traces [29, 30]. Much effort has been invested in developing seedless grapes, including treatment with exogenous gibberellic acid (GA), breeding programs that cross seedless parental genotypes, and obtaining progeny through embryo rescue assisted by in vitro tissue culturing [31]. It was reported that overexpression of grape *VvCEB1*, which encodes a helix–loop–helix transcription factor, affected embryo development and increased cell size [32]. Moreover, *VvAGL11*, a MADS-box seed identity gene, has been proposed as playing a role in stenospermocarpy, and has been suggested as a candidate for use in marker-assisted selection [33]. In addition, five MADS-box genes (*VvMADS28*, *VvMADS39*, *VvMADS44*, *VvMADS45* and *VvMADS46*) were reported to show different expression patterns during seed development between seeded and seedless grape cultivars, indicating a potential role in seed development [34]. It was proposed that parthenocarpy in the seedless somatic grapevine variant, Corinto bianco, may be caused by the absence of a mature macrogametophyte, probably due an arrest in meiosis coupled with fertilization-independent fruit growth [35]. Finally, a transcriptome analysis during berry development provides insights into co-regulated and altered gene expression between a seeded wine grape variety and its seedless somatic variant [36]. Despite all the progress mentioned above, the molecular and genetic mechanisms controlling grape seed size remain unidentified.

**Fig. 1:**
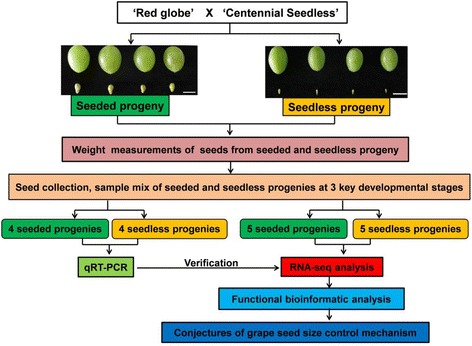
Overview of the experimental design used in this study. Scale bars are 1 cm

The release of the V. vinifera PN40024 genome (12X) sequence [37], has substantially facilitated whole-genome grape transcriptome analysis and functional gene prediction. In this current study, we performed a comprehensive transcriptome analysis to elucidate the molecular mechanisms underlying the seedlessness trait (Fig. 1). This involved a comparative transcriptome analysis of the seeds of seeded and seedless progeny, derived from the seeded maternal parent ‘Red Globe’ (*V. vinifera*) and the seedless paternal parent ‘Centennial seedless’ (*V. vinifera*); an approach that was designed to minimize genetic background differences. The identification of differentially expressed genes (DEGs), and analyses of their putative biological functions and key pathways that predominated in the different phenotypes, enhances the current understanding of grape seed development and sheds light on the possible mechanism by which grape seed size is controlled.

## Results and discussion

### Changes in seeds at different developmental stages of seeded and seedless grapes progeny

**Fig. 2:**
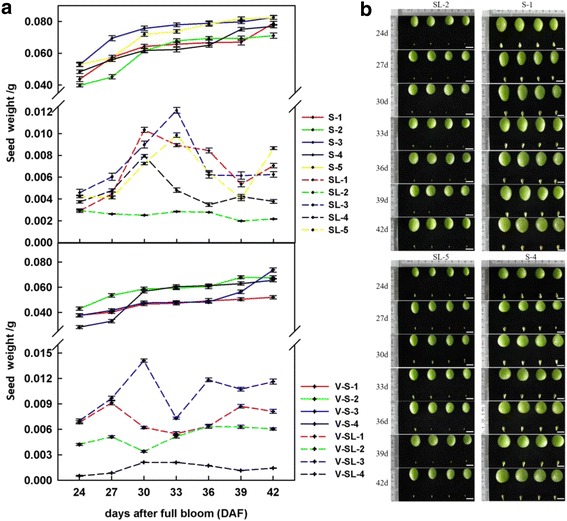
Changes in seed weight and shape in seeded and seedless progeny. **a** Weight of seeds from seeded and seedless progeny. Vertical bars indicate standard errors. S, ‘seeded progeny used for RNA-Seq; SL, seedless progeny used for RNA-Seq; V-S, seeded progeny used for verification; and V-SL, seedless progeny used for verification. **b** Seeds collected for shape change analysis. Photos are representative of seeds and berries at each stage. Scale bar are 1 cm

Seeds of seeded and seedless grape progeny derived from the seeded maternal parent, ‘Red Globe’ (*V. vinifera*) and the seedless paternal parent, ‘Centennial seedless’ (*V. vinifera*), were collected at 24, 27, 30, 33, 36, 39 and 42 DAF to characterize changes and differences between the two (Fig. 2). Seed weight and size of the seeded progeny were significantly greater compared to those of the seedless progeny, and their weight continued to increase with time, while the seed weight of the seedless progeny showed an initial peak and then a decrease, followed by another increase before reaching a constant value. Based on the weight change of seeds in seedless progeny, 3 stages (initial stage, stage with the highest weight, and stage with the lowest weight) were chosen as key developmental stages.

### Identification and expression patterns of DEGs indicate the involvement of TFs and TRs in grape seed development

Approximately 90 % of the cleaned reads could be mapped to the reference *V. vinifera* PN40024 genome (Additional file 1: Table S1). Correlation coefficients of the transcriptome profiles were 0.96 between each set of biological replicates (Additional file 2: Table S2), indicating high reproducibility of our RNA-Seq data.

**Fig. 3:**
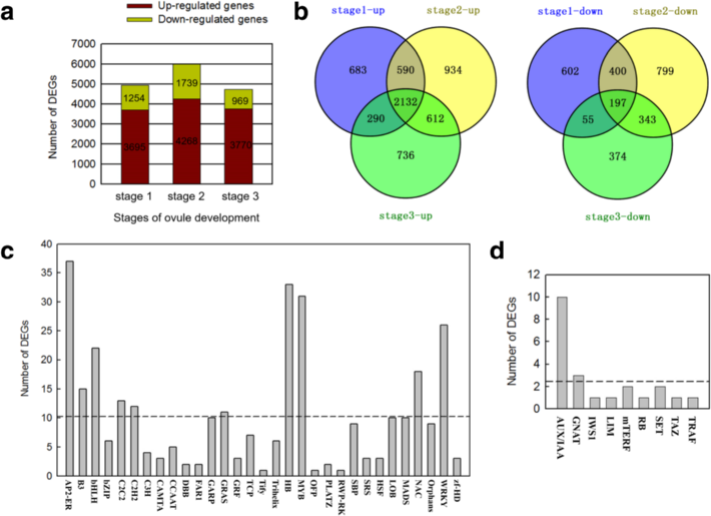
Comparison of gene expression at three seed developmental stages. **a** Number of differentially expressed genes (DEGs, *P* value ≤ 0.05 and fold-change ≥ 2.0) between seedless and seeded grape seed samples at three developmental stages. **b** Venn diagram showing the relationship between up-regulated and down-regulated DEGs identified in each seed developmental stage in seedless progeny compared to seeded progenies. **c** Number of DEGs in different transcription factor families. Dashed lines represent the average number of DEGs in each family. **d** Number of DEGs in different transcription regulator families

Based on seed weight change (Fig. 2a), three key stages (initial stage, stage with the highest weight, and stage with the lowest weight) were chosen. A total of 6,607 DEGs were identified (Additional file 3: Table S3), at all three developmental stages, the numbers of genes up-regulated in seedless (SL) progenies compared to seeded (S) progenies (3,695, 4,268 and 3,770 in stages 1, 2 and 3, respectively) were higher than the numbers of down-regulated genes (1,254, 1,739 and 969 in the same respective stages) (Fig. 3a), and the number of DEGs was highest at stage 2. A total of 2,132 up-regulated and 197 down-regulated genes (SL/S) were common to all three stages (Fig. 3b). We extracted 318 transcription factors (TFs) and 22 transcription regulators (TRs) from the DEGs identified at the three developmental stages, further divided them into 31 TF and 9 TR families. The majority of the TF encoding DEGs were members of the AP2/EREBP family (11.6 %), followed by the HB family (10.4 %), the MYB family (9.8 %), the WRKY family (8.2 %), the BHLH family (6.9 %), the NAC family (5.7 %), the C2C2 family (4.1 %), the C2H2 family (3.8 %) and the GRAS family (3.5 %) (Fig. 3c). Most of the differentially expressed TR genes belonged to the AUX/IAA family (45.5 %), followed by the GNAT family (13.6 %) (Fig. 3d). Most of the TF DEGs showed an up-regulated expression in the seedless progeny compared to the seeded progeny, although some DEGs identified in the C2H2, MYB, LOB and MADS-box families were down-regulated (SL/S) at all three developmental stages (Additional file 4: Figure S1). Likewise, most DEGs identified as TRs were expressed at higher levels in the seedless progeny compared to the seeded ones; especially those in the AUX/IAA and GNAT families (Additional file 5: Figure S2). As previous studies reported, many TFs and TRs play important roles in seed development in wide range of plant species [5]. For example, *TTG2* (*TRANSPARENT TESTA GLABROUS 2*), a WRKY gene, was found to be related to *A. thaliana* seed coat and endosperm development [16, 38]. Moreover, *NAM* (*NO APICAL MERISTEM*), *CUC1* (*CUP-SHAPED COTYLEDON*) and *CUC2*, all NAC genes, have been reported to be involved in regulating secondary cell wall biosynthesis [39, 40]. Many reports have indicated that MADS-box genes play critical roles in ovule, seed and flower development [34, 41, 42] and the expression of *VvMADS28*, *VvMADS39 and VvMADS45* in this current study was consistent with previous analysis of seeds from multiple seeded and seedless grape cultivars [34]. Additionally, TFs such a GRAS and HB are involved in GA and ABA signal transduction, and TRs such as AUX/IAA are important in auxin regulation [43]. In our study we identified examples of all the above mentioned seed-related TFs and TRs that were differently expressed during seed development between seeded and seedless progeny, suggesting an association with the seedless phenotype.

### Comparison of significantly enriched pathways reveals possible pathways influencing grape seed development

**Fig. 4:**
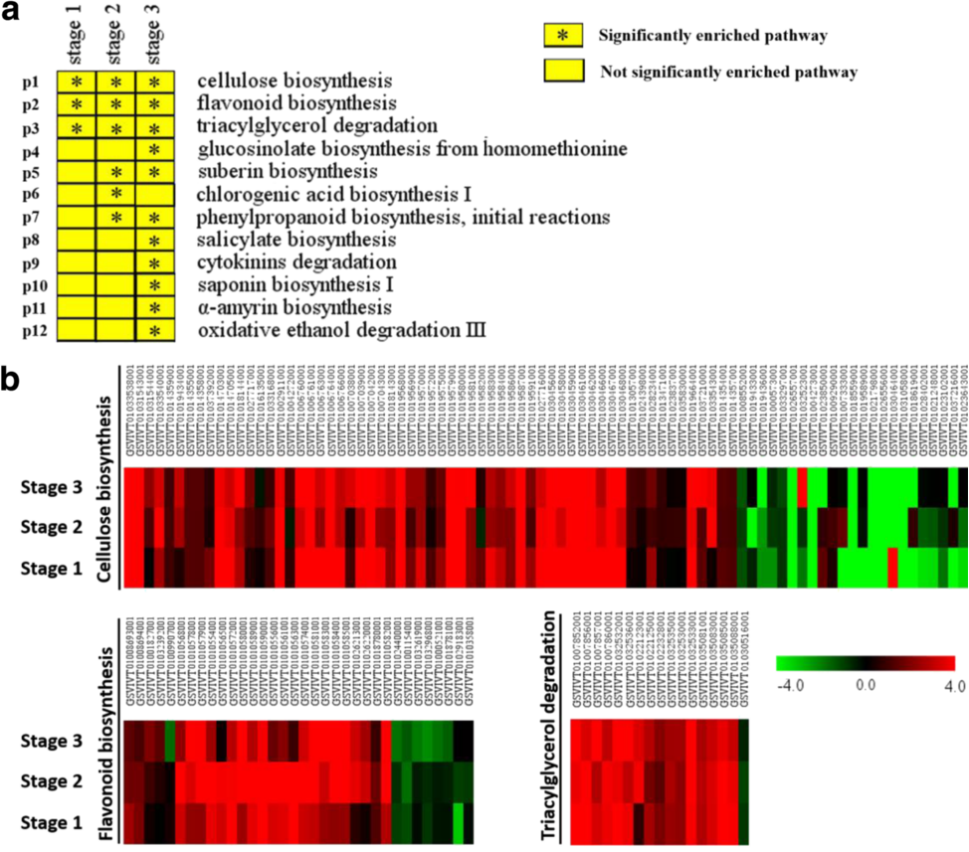
Pathway enrichment analysis of seeded and seedless grape seeds at three developmental stages. **a** Significantly enriched pathways at different seed developmental stages. Squares with ‘*’ indicate significantly changed pathways with P-value less than 0.05. **b** Expression profiles of genes in selected pathways (Log2-transformed fold-change of seedless reads per kilobase of exon model per million mapped reads (RPKM) relative to the seeded RPKM). All the genes were significant DEGs at least at one developmental stage. The maximum/minimum value was set to ±4.0

An analysis using Plant MetGenMAP [44] indicated that a total of 12 molecular pathways were significantly enriched in at least one developmental stage, with 3 pathways being significantly enriched in all three stages (Fig. 4a). These 12 pathways were associated with numerous and diverse metabolic processes, including the biosynthesis or degradation of hormones, sugars, lipids, and secondary metabolites. Three pathways were significantly enriched at stage 1, 6 at stage 2, and 11 at stage 3, indicating an increasing number of differing metabolic processes between seedless and seeded progeny during seed development. Three pathways, annotated as cellulose biosynthesis, flavonoid biosynthesis, and triacylglycerol degradation, were significantly enriched at all three developmental stages, and most of the DEGs involved in the three pathways were up-regulated (SL/S) (Fig. 4b). Genes associated with suberin biosynthesis and the initial reactions in the phenyl propanoid pathway were significantly enriched at both stages 2 and 3, while pathways such as salicylate biosynthesis, cytokinin degradation, and oxidative ethanol degradation were only significantly enriched at stage 3, and chlorogenic acid biosynthesis I only at stage 2. Collectively, these results suggested that pathways related to seed coat and cell wall development, flavonoid biosynthesis, lipid metabolism and hormonal balance may be involved in grape seed development.

### Comparison of enriched gene ontology (GO) terms indicates possible factors contributing to seed abortion

To gain further insight into the functional significance of the identified DEGs, they were classified according to the GO terms in the ‘biological process’ , ‘molecular function’, and ‘cellular component’ categories (Additional file 6: Figure S3 and Additional file 7: Table S4). Overall, most DEGs were up-regulated (SL/S) and there was high representation in the ‘cellular component organization’ , ‘biosynthetic process’ , ‘metabolic process’, ‘response to stress’ , ‘transport’ , ‘protein modification process’ and ‘transcription’ groups of the ‘biological process’ category at all three stages. This was also true for the ‘hydrolase activity’ , ‘kinase activity’ , ‘transferase activity’ , ‘protein binding’ , ‘catalytic activity’ and ‘nucleotide binding’ in the ‘molecular function’ category, and ‘membrane’ , ‘plasma membrane’ , ‘cytoplasm’ and ‘nucleus’ in the ‘cellular component’ category.

**Fig. 5:**
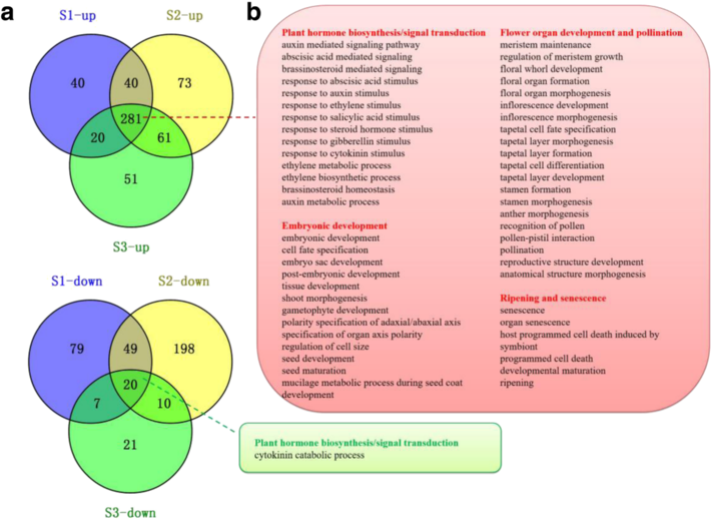
Gene ontology (GO) analysis of seeded and seedless grape seeds at different developmental stages. **a** Venn diagram showing the relationship of GO terms significantly enriched in up- and down-regulated DEGs (SL/S) which were identified at each developmental stage. **b** List of common GO terms significantly enriched in up- and down-regulated DEGs (SL/S) which were involved in ‘plant hormone biosynthesis/signal transduction’, ‘flower and embryonic development’ and ‘ripening and senescence’ at three seed developmental stages. The red and green squares represent GO terms associated with up- and down-regulated DEGs (SL/S), respectively

Amongst the annotated biological processes, some were related to plant growth and development, stimulus and signal transduction, glucolipid metabolism and energy, differentiation, mitosis, endoreduplication, and epigenetic regulation. All the biological process mentioned above shed light on the differences in multiple metabolic processes between seedless and seeded grape progeny during seed development. In the ‘biological process’ category, common significantly enriched GO terms in the up- or down-regulated DEGs (SL/S) during seed development are shown in Fig. 5 and Additional file 8: Table S5. A total of 281 and 20 GO terms, which were separately enriched in the up- and down-regulated DEGs (SL/S), were common at all three developmental stages (Fig. 5a). Those GO terms enriched in up-regulated DEGs (SL/S) were mainly related to growth and development, and were mostly classified into four groups (‘plant hormone balance and signal transduction’, ‘embryonic development’ , ‘flower organ development and pollination’ , and ‘ripening and senescence’), while the ‘cytokinin catabolic process’ was the only GO term enriched in down-regulated DEGs (SL/S) in the ‘growth and development’ category (Fig. 5b). The results were consistent with the pathway analysis (Fig. 4), indicating that the GO terms related to growth and development and hormone metabolism differed significantly between the seeds of seedless and seeded progeny. We concluded that the GOs enriched in up-regulated DEGs (SL/S), which were related to hormone metabolism, seed coat polarity establishment, reproductive structure development, and programmed cell death, are potential factors contributing to seed abortion in the seedless progeny.

### Hormone content differences between seeded and seedless progeny and their potential relationship with grape seedlessness

**Fig. 6:**
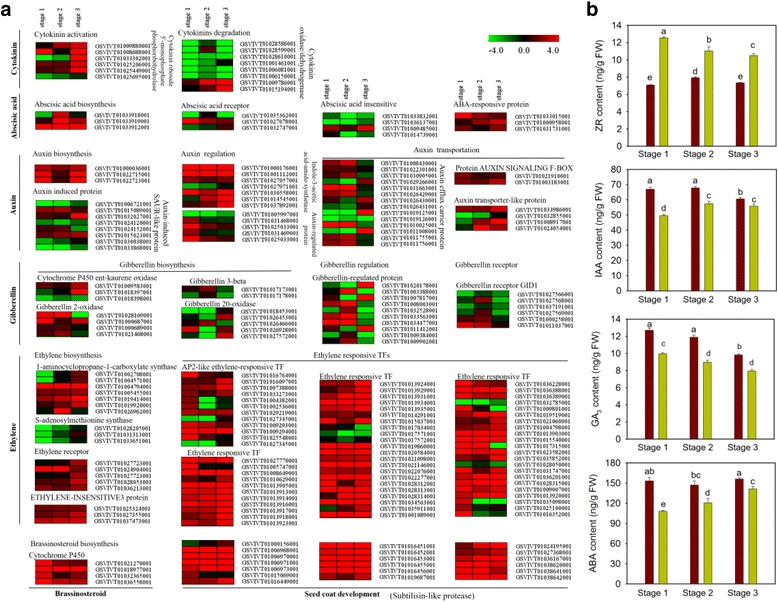
Differences in hormone level and hormone-related DEGs between seeded and seedless progeny. **a** Expression profiles of differentially expressed genes (DEGs) involved in ‘plant hormone homeostasis and signaling pathways’ and ‘seed coat development’ (Log2-transformed fold-change of seedless reads per kilobase of exon model per million mapped reads (RPKM) relative to seeded RPKM) at three developmental stages. All genes were significant DEGs at least at one developmental stage. The maximum/minimum value was set to ±4.0. **b** Hormone content of seeds in seeded and seedless progeny at three developmental stages. Bars represent standard errors of the mean. Different letters indicate statistically significant differences (Dunn’s test; *P* < 0.05)

Seed development is known to result from the coordinated regulatory actions of multiple hormones [45]. We measured the content of four hormones and analyzed the expression profiles of DEGs involved in hormone homeostasis (Fig. 6).

Higher level of ZR were found in seeded progeny at all three developmental stages (Fig. 6b), and most of the DEGs involved in cytokinin activation were up-regulated (SL/S), especially at stage 3, while most Cytokinin oxidases/dehydrogenases (CKX) genes were expressed at lower levels at all three developmental stages in the seedless progeny (Fig. 6a), indicating a feedback regulation of CTK homeostasis. Besides, we observed that the content of auxin is higher in seedless progeny (Fig. 6b). Accordingly, the DEGs involved in auxin synthesis and F-box receptor proteins were up-regulated (SL/S) at all three stages (Fig. 6a), as were the AUX/IAA transcriptional repressors (Additional file 5: Figure S2), which are central to the auxin response [43], in accordance with the higher content of auxin in seedless progeny. It has been hypothesized that the growth of the ovary is blocked prior to pollination and that auxin is involved in de-repression of ovary growth after fertilization [46]. Another class of hormones, the brassinosteroids (BRs) play prominent roles in plant cell elongation and differentiation and interact synergistically with auxins [47]. We identified cytochrome P450, a key component of BR synthesis as being expressed at higher levels in all three developmental stages of the seedless progeny, indicating higher levels of BR in seedless progeny. In addition, the content of GA_3_ was measured and found to be higher in seedless progeny than seeded progeny (Fig. 6b). Accordingly, gibberellin 2-oxidase (GA2ox), which contributes to GA catabolism, was mostly up-regulated (SL/S) (Fig. 6a). Furthermore, genes encoding DELLA proteins, which act as repressors of GA signaling, and members of the GRAS family, almost all showed higher expression in the seedless progeny at all three stages (Additional file 4: Figure S1), indicating a feedback regulation of GA homeostasis. All in all, as BR and GA_3_ were all hormones promoting cell division and plant growth enlargement [48], and there is evidence that application of BR induces parthenocarpic growth in cucumber, accompanied by active cell division [49], thus we wonder whether the higher content of GA_3_ and auxin in seedless progeny also caused increased cell division and led to grape seed abortion.

On the other hand, ABA and ethylene are known to promote ripening and senescence [43], and from our analyses that ABA level was higher in seedless than in seeded progeny during seed development (Fig. 6b). Accordingly, we observed substantial numbers of up-regulated DEGs (SL/S) related to ABA biosynthesis and responses in the seedless progeny (Fig. 6a). Moreover, it is well established that the hormone abscisic acid (ABA) regulates seed coat development during early seed filling [50]. We observed that DEGs related to seed coat development were almost all up-regulated (SL/S), consistent with the higher content of ABA in seedless progeny. The hypothesis that increased production of ABA promotes seedless progeny seeds ripening and senescence and caused seed abortion needs to be tested in future. Finally, we identified DEGs associated with the gaseous hormone ethylene, most of which were annotated as being involved in the biosynthesis and signal transduction processes and found to be up-regulated (SL/S). Taken together we inferred that differences in hormonal content and their corresponding hormonal-related genes expression between the seedless and seeded progeny may contributed to seed abortion in the former.

### Transcriptional dynamics analysis of DEGs and a Map of core molecular processes underlying seed development in seedless and seeded progeny

**Fig. 7:**
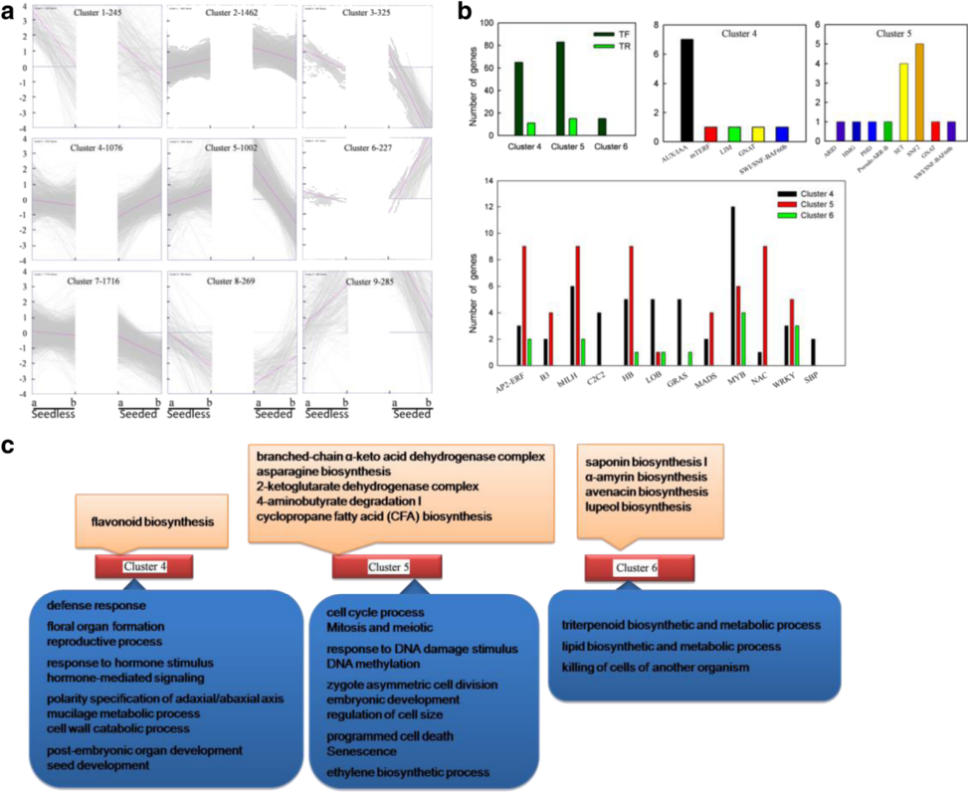
Differentially expressed genes (DEGs) with different expression patterns during grape progeny seed developmental stages. **a** Clustering of the expression profiles of DEGs from both seeded and seedless progeny at different seed developmental stages. Clustering was performed using the k-means method and 9 clusters were chosen for further analysis of transcriptional patterns. The number of genes in each cluster is listed after the cluster IDs. The ‘a’ at the X-axis stands for ‘stage 2 versus stage 1’ and ‘b’ stands for ‘stage 3 versus stage 2’. The Y-axis indicates the Log2-transformed fold-change of relative reads per kilobase of exon model per million mapped reads (RPKM) among the developmental stages (stage 2 versus stage 1 and stage 3 versus stage 2). The maximum/minimum value was set to ±4.0. **b** Transcription factors and regulators identified in selected clusters. **c** Pathway and Gene Ontology (GO) analysis of each cluster. The orange squares represent significantly changed pathways and the blue squares represent GO terms

To investigate major transcriptional dynamics associated with seed development, a K-mean clustering approach was used to group genes with similar expression profiles during seed developmental. All the 6,607 DEGs identified previously were then divided into 9 co-expression clusters (Fig. 7a), of which 3 were chosen for further analysis on the basis of their different gene expression tendency during seed development of seeded and seedless progenies. The expression of DEGs in Cluster 4 (1,076 genes) and Cluster 6 (227 genes) was first up-regulated (stage 2 versus stage 1) and then down-regulated (stage 3 versus stage 2) in the seedless progeny, while the opposite pattern was detected in seeded progeny. This tendency was completely reverse. However, the magnitude of the change was greater in Cluster 6. Similarly, for Cluster 5, genes in the seedless progeny showing down-regulation from stage 1 to stage 2 and then up-regulation from stage 2 to stage 3, while the opposite was true for the seeded progeny. The expression patterns of all the DEGs in Clusters 4, 5 and 6 were stage-associated, with the highest or lowest expression at stage 2 and an opposite expression pattern in the seeded and seedless progeny.

TF and TR genes that fell within Cluster 4, 5 and 6 were analyzed further (Fig. 7b). Cluster 5 contained most TR and TF genes, while Cluster 4 had a high representation of genes belonging to the AUX/IAA TR family, while most DEGs in Cluster 5 belonged to the SNF2 and SET TR families, indicating the involvement of distinct regulatory networks during seed development in the two types of progeny. TFs from the three clusters were classified into 12 families, with the largest number belonging to the MYB family, followed by bHLH and AP2/ERF families. For 7 TF families (AP2/ERF, B3, bHLH, HB, MADS-box, NAC and WRKY) the number of DEGs in Cluster 5 was higher than in Clusters 4 or 6, although no C2C2, GRAS and SBP TF members in Cluster 5.

The DEGs from Clusters 4, 5 and 6 were further analyzed for differences in GO terms and pathways (Fig. 7c). In Cluster 4, significantly changed pathways included ‘flavonoid biosynthesis’ , while the terms ‘tricarboxylic acid cycle (TCA)’ and ‘lipid metabolism’ were enriched in Cluster 5, as were pathways related to ‘terpenoid biosynthesis’ in Cluster 6, indicating that theses pathways were stage-associated and may be key factors related to seed development differences between seeded and seedless progeny. In addition, DEGs in each cluster were also found to be enriched in different groups within the ‘biological processes’ category. In Cluster 4, GO terms related to ‘defense response’ , ‘reproduction’, ‘hormone regulation’ , ‘seed coat development’ and ‘seed development’ were enriched, while the groups ‘cell cycle’ , ‘DNA methylation’ , ‘embryonic development’ , ‘cell death’ , ‘senescence’ and ‘ethylene biosynthesis’ were enriched in Cluster 5. And Cluster 6 was enriched in the terms ‘triterpenoid’ , ‘lipid’ and ‘cell death’. This comparative analysis of DEGs during seed development between seeded and seedless progenies takes into account both their transcriptional dynamics and their associated molecular processes. The results revealed that these stage-associated pathways and biological processes were mainly related to hormone homeostasis, seed coat development, primary and secondary metabolism, epigenetic regulation, cell cycle, and reproductive development, all of which were important in seed development, indicating that these differences may be the reason causing seed abortion.

### Validation of RNA-Seq results

**Fig. 8:**
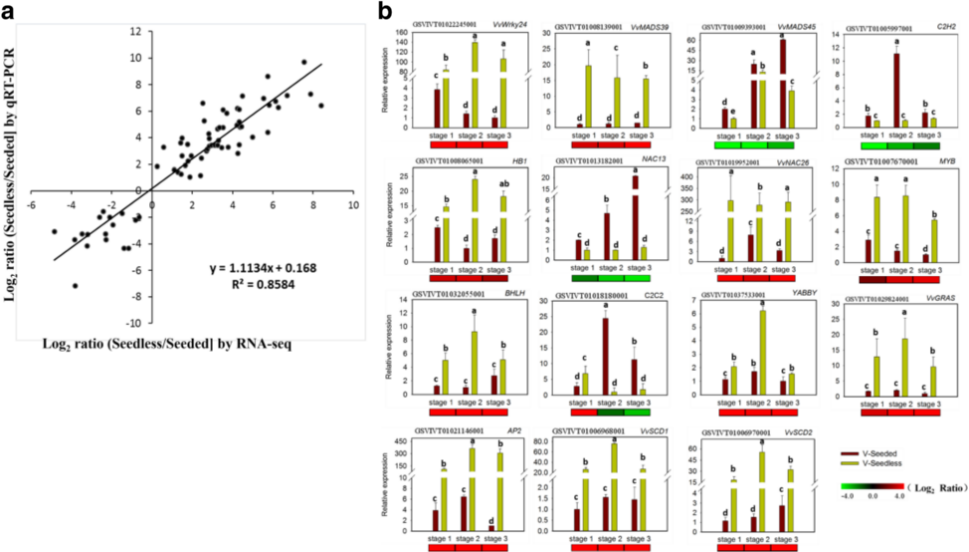
Verification of RNA-Seq results by qRT-PCR. **a** Correlation between RNAseq and qRT-PCR. The Pearson's correlation coefficient between relative expression levels is shown. **b** Fifteen genes were selected for validation of the RNA-Seq data by qRT-PCR. Heat maps under the histograms show a comparison of gene expression in the seeds of seedless and seeded progeny (Log2-transformed fold-change of seedless reads per kilobase of exon model per million mapped reads (RPKM) relative to the seeded RPKM at three developmental stages based on RNA-Seq results. The maximum/minimum value was set to ±4.0. ‘V-Seeded’ represents ‘seeded progeny used for verification’ and ‘V-Seedless’ represents ‘seedless progeny used for verification’

To validate the RNA-Seq based DEG data, we quantified the expression of 30 DEGs related to seed development using quantitative real-time PCR (qRT-PCR) analysis in samples ‘Seeded’ and ‘Seedless’ which were previously used for RNA-Seq (Additional file 9: Figure S4). A correlation graph of fold change values from both methods were made (Fig. 8a), indicating the results were highly consistent. All the selected DEGs included genes involved in flower and seed identity like *VvMADS39*, *VvMADS45*, *VvSTM* and *AP2*, genes related to seed coat development like *VvNAC26*,*VvNAC86*,*SCD1* and *SCD2*,genes related to endosperm development and epigenetic regulation like *VvPHE1*, *VvDME* and *VvDDM1*, and genes associated with hormone balance like *GRAS*, *GH3-1* and *GH3-9*. Besides, the expression patterns of two MADS-box genes *VvMADS45* (GSVIVT01009393001) and *VvMADS39* (GSVIVT01008139001) were found to be consistent with those presented in a previously published study [34]. Moreover, based on the above mentioned results, 15 out of the 30 DEGs were further selected and subjected to qRT-PCR validation using samples ‘V-Seeded’ (seed mixtures from another 4 seeded progeny) and ‘V-Seedless’ (seed mixtures from another 4 seedless progeny) (Fig. 8b). The results of the qRT-PCR analysis were also consistent with our RNA-seq, which to some extent enhanced the reliability of our data in a relatively wide range.

### Expression network Map of grape seed-related DEGs reveals possible mechanisms controlling grape seed size

**Fig. 9:**
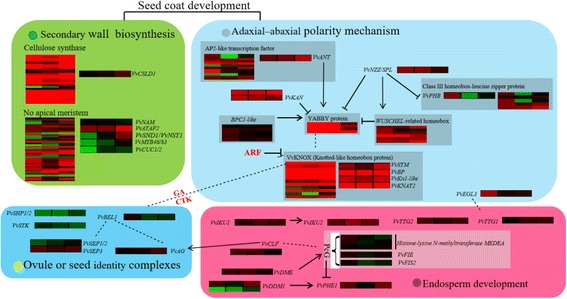
Model of the interaction of genes critical for grape seed development. Based on existing research, differentially expressed genes (DEGs) with the highest homology to key *A. thaliana* seed-related genes are shown with their expression profiles (Log2-transformed fold-change of seedless reads per kilobase of exon model per million mapped reads (RPKM) relative to the seeded RPKM at three seed developmental stages. All the genes were significant DEGs at least at one developmental stage. The maximum/minimum value was set to ±4.0

Pathways and regulators affecting either ovule or seed development in *A. thaliana* have been well studied [41] and provide a valuable reference for similar studies in other plant species. Based on previously reported data [5, 38, 41, 45], we identified candidate seed-related genes by searching our DEGs with homologous genes of reported ovule- or seed-related genes in *A. thaliana* (Additional file 10: Table. S6). A map of potential grape seed-related DEGs with their expression pattern was drawn on four aspects including hormone regulation, seed coat development, endosperm development, and ovule and seed identity complex formation (Fig. 9). The networks were supported by multiple lines of evidence, including the expression profiles of DEGs, enriched GO terms and pathways, and inferred association with hormone regulation. In our results, most of the DEGs with homology to *A. thaliana* second wall-related genes, such as *CSLD1*, *NACs* (*NAM*, *ATAF1*/*2*, *CUC2*), *MYB46* and *MYB83* [51–54], were up-regulated (SL/S). Besides, as the establishment of adaxial–abaxial polarity is a prerequisite of seed coat development [26, 38, 50], we found that most of grape polarity establishment-related DEGs were up-regulated (SL/S) during seed development (Fig. 9), in agreement with the observation that seed coat development genes were almost all up-regulated (SL/S) (Fig. 6a). This was also the case for genes associated with the biosynthesis of ABA (Fig. 6a), which has been reported to be associated with seed coat formation during early seed filling [55]. Moreover, pathways related to seed coat development, like ‘cellulose biosynthesis’ and ‘suberin biosynthesis’ , were found to be significantly enriched (Fig. 4). Given the importance of the seed coat in the determination of seed quality traits, such as size, composition and permeability, as well as hormonal regulation [50], taken all the mentioned results together, we suggest the differences in the seed coat development may contribute to seed phenotype differences.

Another factor that influences seed size is the development of the endosperm, which has been shown to be under epigenetic control [26, 56, 57], leading to our analysis of grape DEGs which were homologous to *A. thaliana* epigenetic regulation related genes. In our results, we observed that most *VvPcG* genes were expressed at lower levels (SL/S) during seed development in the seedless progeny, especially at stage 2 and 3, than in the seeded progeny, while conversely, the expression of endosperm-related *VvIKU1*/*2*, *VvTTG2* and *VvTTG1* was higher (SL/S)*.* From previous reported *A. thaliana* epigenetic regulation mechanism[1] [57], PcG proteins can regulate embryo and endosperm proliferation by repressing the expression of type I MADS-box gene *PHE1*, while *DECREASE IN DNA METHYLATION1* (*DDM1*) act antagonistically. And the high *PHE1* expression in *A. thaliana* is primarily responsible for the *mea* seed-abortion phenotype [1]. In our results, it seems that the model still applies: the expression of *VvPHE1* was up-regulated (SL/S), probably resulting from the combination of down-regulated expression of *VvPcG* and up-regulated expression of *VvDDM1* (SL/S) (Fig. 9). In this way, we conclude that control of seed development is a complex developmental event influenced by both genetic and epigenetic processes, and propose that high *PHE1* expression may be an important contributing factor in the seed-abortion phenotype of seedless progeny.

We also observed that among the potential ovule and seed identity genes, the expression of *VvSHP1*/*2*, *VvSTK*, *VvBEL1* and *VvSEP1*/*2*, was lower during seed development in the seedless progeny than in the seeded progeny (Fig. 9), suggesting these genes may be key factors related to seed abortion. Overall, in the network analysis, we noted that *VvBEL1* and *VvAG* were connected with *VvKNOX* genes, which were related to seed coat development, as well as to *VvCLF*, which was related to endosperm development, indicating that ovule and seed identity genes may be affected by both seed coat and endosperm development. Taken together, we found evidence for commonalities in the molecular mechanisms underlying in *A. thaliana* seed development and the development of grape seeds. This model we proposed to some extent showed light on grape seed development study, more efforts are still needed to test our hypothesis.

## Conclusions

To summarize, to investigate possible mechanisms leading to the seedlessness phenotype in grape, we performed an integrative transcriptomic analysis of seed development in seedless and seeded grape progeny. The RNA-Seq data was used to identify DEGs, as well as GO terms and pathways that distinguished in the seeded and seedless progeny and revealed a set of candidates that were associated with seed development and their regulation. Major differences were focused on aspects of hormone regulation, the development of the seed coat and endosperm, and in the formation of ovule and seed identity complexes. Overall the data provides insights into the possible molecular mechanism influencing grape seed size, which is of great importance for both basic research and future seedless grape breeding.

## Methods

### Plant material

Cross-progeny populations from the seeded maternal parent ‘Red Globe’ (*V. vinifera*) and the seedless paternal parent ‘Centennial seedless’ (*V. vinifera*) were developed in 2009 and planted in 2011 as family groups at the Zhengzhou Fruit Institute, Zhengzhou, China. A total of 65 progeny were obtained: 31 seedless and 34 seeded. In 2014, 9 seedless progeny and 9 seeded ones, selected at random from the populations, were grown under similar growth condition (based on previous observation) and used as experimental material.

### Sample collection and seed weight measurements

Previous studies [34, 58] have reported that seed weight of seedless grapes usually begin to decrease at 27 ~ 33 days after full bloom (DAF), and so seeds from 9 seedless and 9 seeded grape progeny were collected at 24, 27, 30, 33, 36, 39 and 42 DAF (Additional file 11: Figure S5). Fruits of all progeny individuals were slashed with scalpel, and then seeds were immediately picked out without damage using tweezers and put into centrifuge tubes which were already soaked in liquid nitrogen. At each stage, one hundred seeds from the selected progeny were selected randomly for total weight measurements, and based on the resulting data, three key stages (initial stage, stage with the highest weight, and stage with the lowest weight) were chosen for further analysis. Based on the sample collection stages of the seedless progeny, three stages (24 DAF, 30 DAF and 39 DAF), which were chosen with maximum frequency among seedless progeny, were chosen as key stages of seeded progeny. At each stage, seeds from 5 seedless progeny were mixed in equal weight proportions, resulting in two replicates for RNA extraction and RNA-Seq analysis (Sample ‘Seedless’). The same were applied to the seeded progeny (Sample ‘Seeded’). Seeds from another 4 seedless and 4 seeded progeny at the three stages were collected at the same time and mixed as material to be later used for verification of RNA-Seq results (Sample ‘V-Seedless’ and ‘V-Seeded’). All seed samples were immediately frozen in liquid nitrogen and stored at −80 °C until further use.

### Measurement of endogenous hormones

At each developmental stage, approximately 1 g of seeds from both seeded and seedless progeny pool were collected and frozen in liquid nitrogen, respectively. The samples were crushed using mortar and pestle and extracted with 4 m L 80 % methanol containing 1 % 2, 6-di-tert-butyl-4-methylphenol at 4 °C for 12 hours. After centrifuging at 4000 rpm for 10 min, the supernatant was isolated using solid phase extraction (SPE) with Accu Bond C18SPE cartridge (Agilent technologies Inc., USA), and then dried using nitrogen gas [59]. The residue was used for the subsequent ciELISA. A Multiskan Mk3 (Thermo, USA) was used to measure the reaction product at 490 nm to estimate the specific hormone concentration, which based on the regressive equations between the optical density and the standard hormone concentration. The final concentration of hormones is given as mean of the three replicated samples per each treatment.

### Statistical analysis

One way analysis of variance (ANOVA) on ranks followed by a Dunn’s test was performed using the SPSS 18.0 software (SPSS Inc., Chicago, IL, USA). Different letters above each bar represent statistically significant differences (Dunn’s test; *P* < 0.05).

### RNA extraction and RNA-Seq analysis

Two biological replicates of each pooled seeds sample at each stage were used for the RNA-Seq experiments. Total RNA was extracted using the E.Z.N.A.™ Plant RNA Kit (OMEGA, China), according to the manufacturer’s instructions. The quality and quantity of RNA was assessed by electrophoresis on 1 % agarose gels and by a NanoDrop1000 spectrophotometer (Thermo Scientific, Wilmington, DE, USA), respectively [60]. Strand-specific RNA-Seq libraries were constructed using previously published protocols [61] and sequenced on an Illumina HiSeq 2000 instrument (at the Genomics Resources Core Facility Cornell at Weill Cornell Medical College) operating in the single-end mode and generating reads with length of 101 bp. RNA-Seq reads were first processed to remove Illumina adaptor and low quality sequences using Trimmomatic [62]. The resulting reads were aligned to ribosomal RNA sequences [63] using Bowtie [64] allowing 3 mismatches and those that aligned were discarded. The resulting filtered reads were then aligned to the *V. vinifera* 12 × PN40024 genome [37] using Tophat allowing 2 mismatches [65]. After alignment, the count of mapped reads from each sample was derived and normalized to reads per kilobase of exon model per million mapped reads (RPKM). DEGs at each time point were identified using the DESeq 1.8.3 package [66] with the raw count data. Raw P values were adjusted for multiple testing using a false discovery rate (FDR) [66, 67]. Genes with an FDR < 0.05 and fold-changes > 2.0 were regarded as DEGs. GO and pathway enrichment analysis were performed using Plant MetGenMAP [44]. To reveal the expression patterns of DEGs in the three developmental stages between seeded and seedless progeny, the K-means clustering were performed using Gene-E (https://software.broadinstitute.org/GENE-E/download.html) [68].

### Quantitative RT-PCR analysis

Quantitative real-time RT-PCR was carried out using a Bio-Rad iQ5 thermo cycler (Bio-Rad, Hercules, CA, USA). For each sample, 1 μg of total RNA was converted into cDNA using PrimeScript™ RTase and an oligo dT primer (TaKaRa Biotechnology, Dalian, China) and was subsequently diluted six times with sterile water. Each reaction was carried out in triplicate with a reaction volume of 20 μl containing 0.8 μl each primer (1.0 μM), 1.0 μl of cDNA, 10 μl of SYBR green (TaKaRa Bio Inc.), and 7.4 μl sterile distilled water. The PCR parameters were 95 °C for 30s, followed by 40 cycles of 95 °C for 5 s and 60 °C for 30s. Melt-curve analyses were performed at 95 °C for 15 s and then a constant increase from 60 °C to 95 °C. The grape *ACTIN* gene (GenBank Accession number NC_012010) and *EF1-α* gene (GenBank Accession number NC_012012) as internal reference genes. All primers were designed using Primer5 software and can be found in Additional file 12: Table S7. Relative expression levels were analyzed using the IQ5 software [69] and the geNorm software [70].

## Additional files

Additional file 1: Table S1. Number of reads cleaned and mapped with Tophat of RNA-seq samples. ‘S’ represents ‘stage’. (XLS 19 kb)
Additional file 2: Table S2. Correlation coefficients of transcriptome profiles among RNA-seq samples. ‘S’ stands for ‘stage’. (XLS 19 kb)
Additional file 3: Table S3. List of DEGs identified at 3 developmental stages. (XLS 1796 kb)
Additional file 4: Figure S1. Expression profiles of genes from different transcription factor families. Number one to 31 indicate different transcription factor families. The maximum/minimum value was set to ±4.0. (PNG 1589 kb)
Additional file 5: Figure S2. Expression profiles of genes from different transcription regulator families. The maximum/minimum value was set to ±4.0. (PNG 243 kb)
Additional file 6: Figure S3. Gene Ontology classification of differentially expressed genes between seeds of seeded and seedless grape progeny. (PNG 939 kb)
Additional file 7: Table S4. Functional categorization of differentially expressed grape genes based on Gene Ontology (GO). (XLS 39 kb)
Additional file 8: Table S5. List of common significantly enriched Gene Ontology terms among the DEGs at three stages of seed development between seedless and seeded grape progeny. (XLS 42 kb)
Additional file 9: Figure S4. Verification of RNA-Seq results by q RT-PCR. Thirty genes were selected for validation of the RNA-Seq data by qRT-PCR. ‘Seeded’ represents ‘seeded progeny used for RNA-Seq’, ‘Seedless’ represents ‘seedless progeny used for RNA-Seq’. (PNG 962 kb)
Additional file 10: Table S6.
*Arabidopsis* procedures used to search for the best grapevine homologs genes. (XLS 32 kb)
Additional file 11: Figure S5. Clusters of seeded and seedless grape progeny during sample collecting. ‘S’ represents ‘seeded’ and ‘SL’ ‘seedless’. Representative images are shown for each stage. (PNG 8508 kb)
Additional file 12: Table S7. The primer sequences used for qRT-PCR amplification. (XLS 22 kb)

## Abbreviations

ABA: Abscisic acid
*AG*: *AGAMOUS*
*BEL1*: *BELL1*
BRs: Brassinosteroids
cDNA: Complementary DNA
CKX: Cytokinin oxidases/dehydrogenases
*CUC*: *CUP-SHAPED COTYLEDONS*
DAF: Days after full bloom
*DDM1*: *DECREASE IN DNA METHYLATION1*
DEGs: Differentially expressed genes
FDR: False discovery rate
FIE/ FIS3: FERTILIZATION-INDEPENDENT ENDOSPERM
FIS: FERTILIZATION INDEPENDENT SEED
GA: Gibberellic acid
GA2ox: Gibberellin 2-oxidase
GA20ox: Gibberellin 20-oxidase
GA3ox: Gibberellin 3-beta-dioxygenase
GO: Gene Ontology
*IKU2*: *HAIKU2*
*INO*: *INNER NO OUTER*
*KAN*: *KANADI*
*KNOX*: *Knotted1-like Homeobox*
MEA/FIS1: MEDEA
*MINI3*: *MINISEED3*
MSI1: MULTICOPY SUPRESSOR OF IRA
*NZZ*/*SPL*: *NOZZLE*/*SPOROCYTELESS*
PcG: Polycomb
PCR: Polymerase chain reaction
*PHB*: *PHABULOSA*
ROS: Reactive oxygen species
RPKM: Reads per kilobase of exon model per million mapped reads
SAUR: Small auxin-upregulated RNAs
S: Seeded
*SEP*: *SEPALLATA*
SL: Seedless
*SHP*: *SHATTERPROOF*
*STK*: *SEEDSTICK*
*STM*: *SHOOT MERISTEMLESS*
SWN: SWINGER
TCA: Tricarboxylic acid cycle
TF: Transcription factor
TRs: Transcription regulators
*TTG2*: *TRANSPARENT TESTA GLABROUS 2*
