# Supplementary figures and images for: Transcriptome analyses of seed development in grape hybrids reveals a possible mechanism influencing seed size
Source: BMC Genomics. 2016 Nov 9;17:898. doi: 10.1186/s12864-016-3193-1 (PMC5103508; doi:10.1186/s12864-016-3193-1)

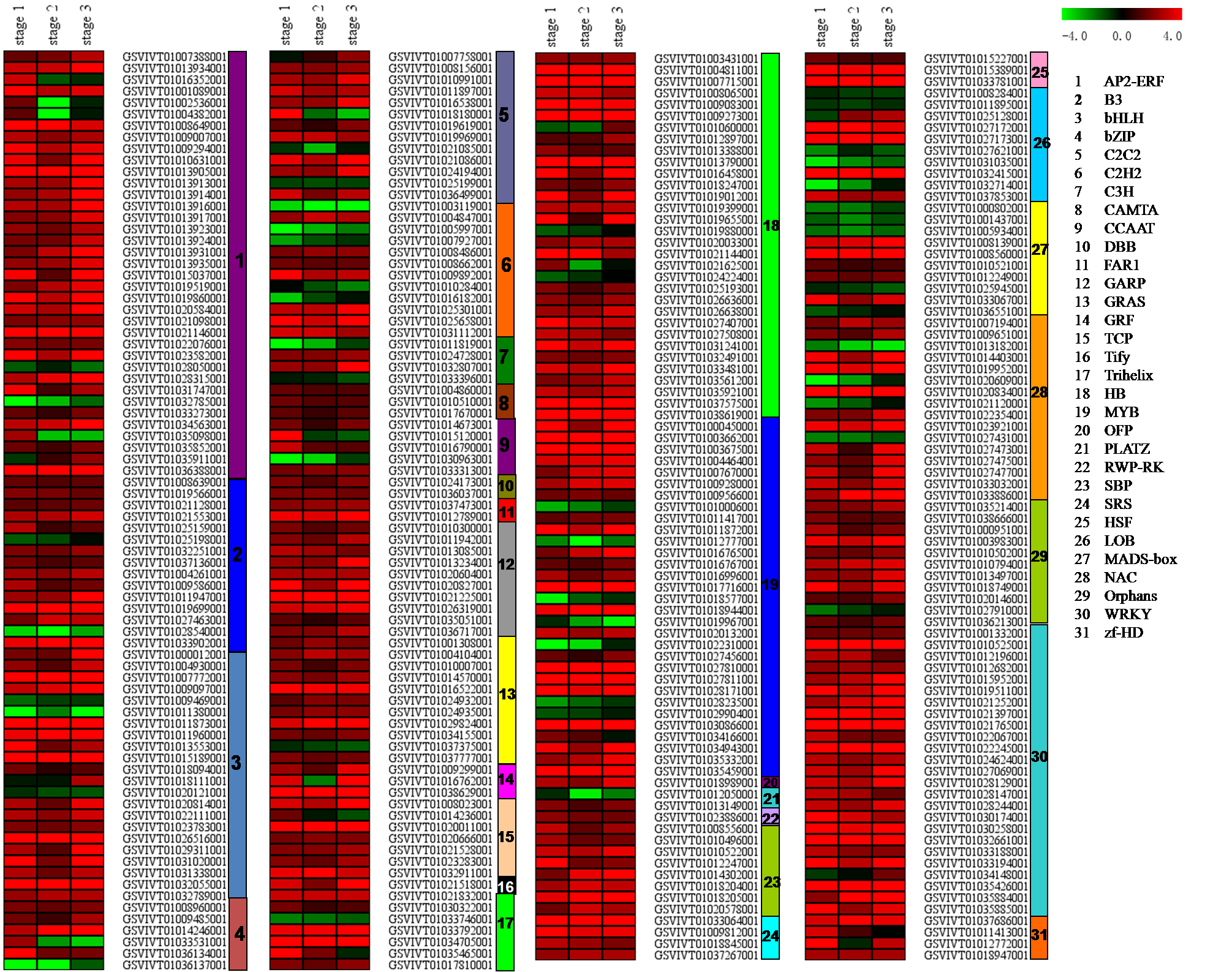

Supplement: Additional file 4: Figure S1. — Expression profiles of genes from different transcription factor families. Number one to 31 indicate different transcription factor families. The maximum/minimum value was set to ±4.0. (PNG 1589 kb) [file 12864_2016_3193_MOESM4_ESM.png]

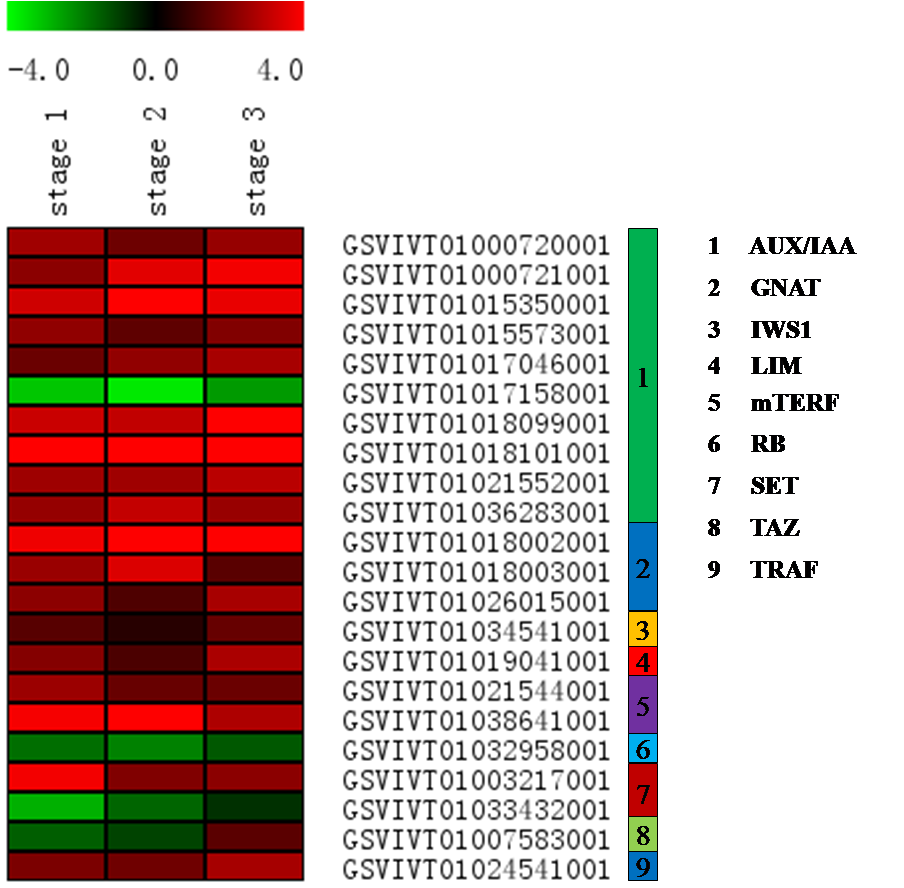

Supplement: Additional file 5: Figure S2. — Expression profiles of genes from different transcription regulator families. The maximum/minimum value was set to ±4.0. (PNG 243 kb) [file 12864_2016_3193_MOESM5_ESM.png]

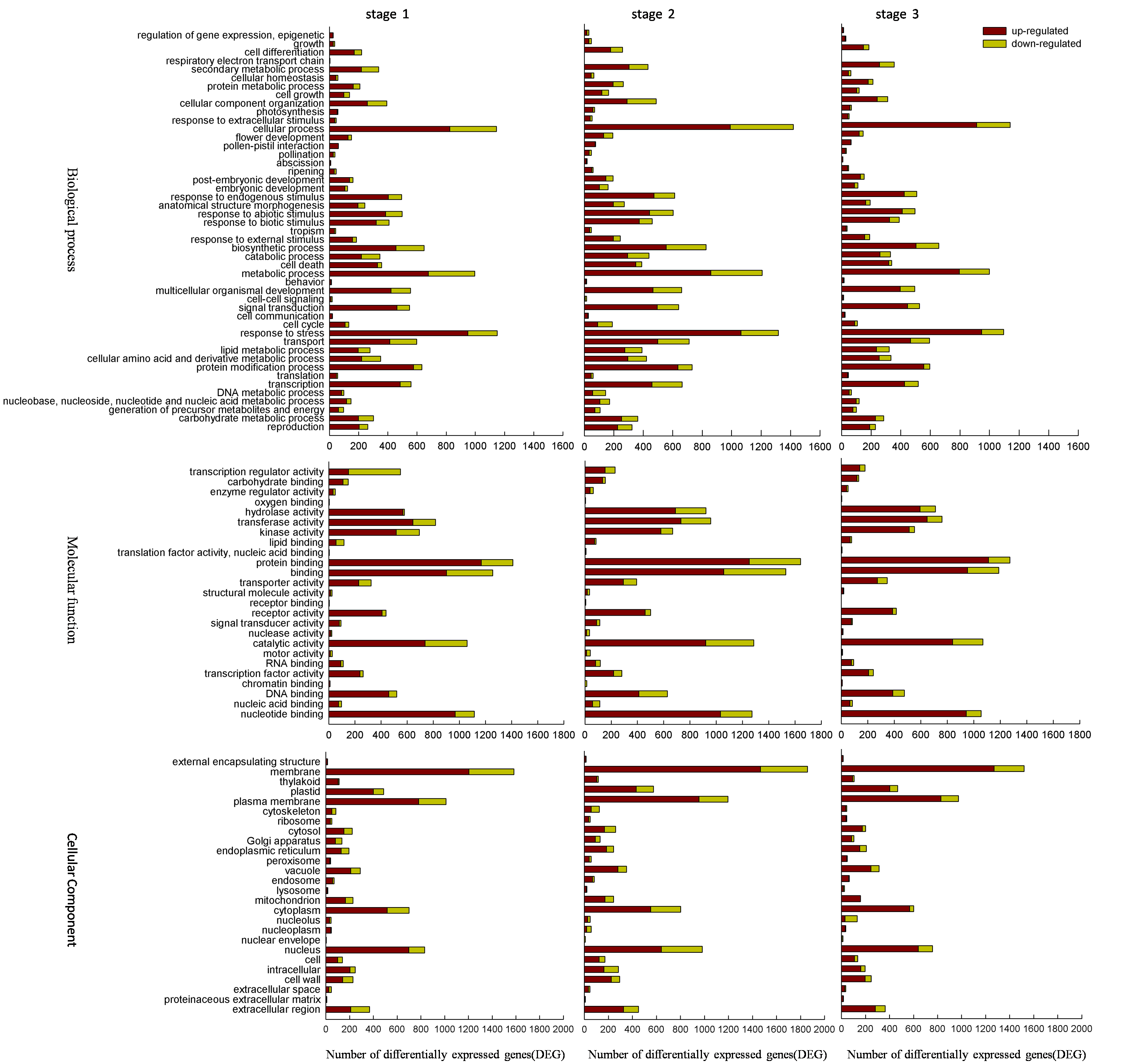

Supplement: Additional file 6: Figure S3. — Gene Ontology classification of differentially expressed genes between seeds of seeded and seedless grape progeny. (PNG 939 kb) [file 12864_2016_3193_MOESM6_ESM.png]

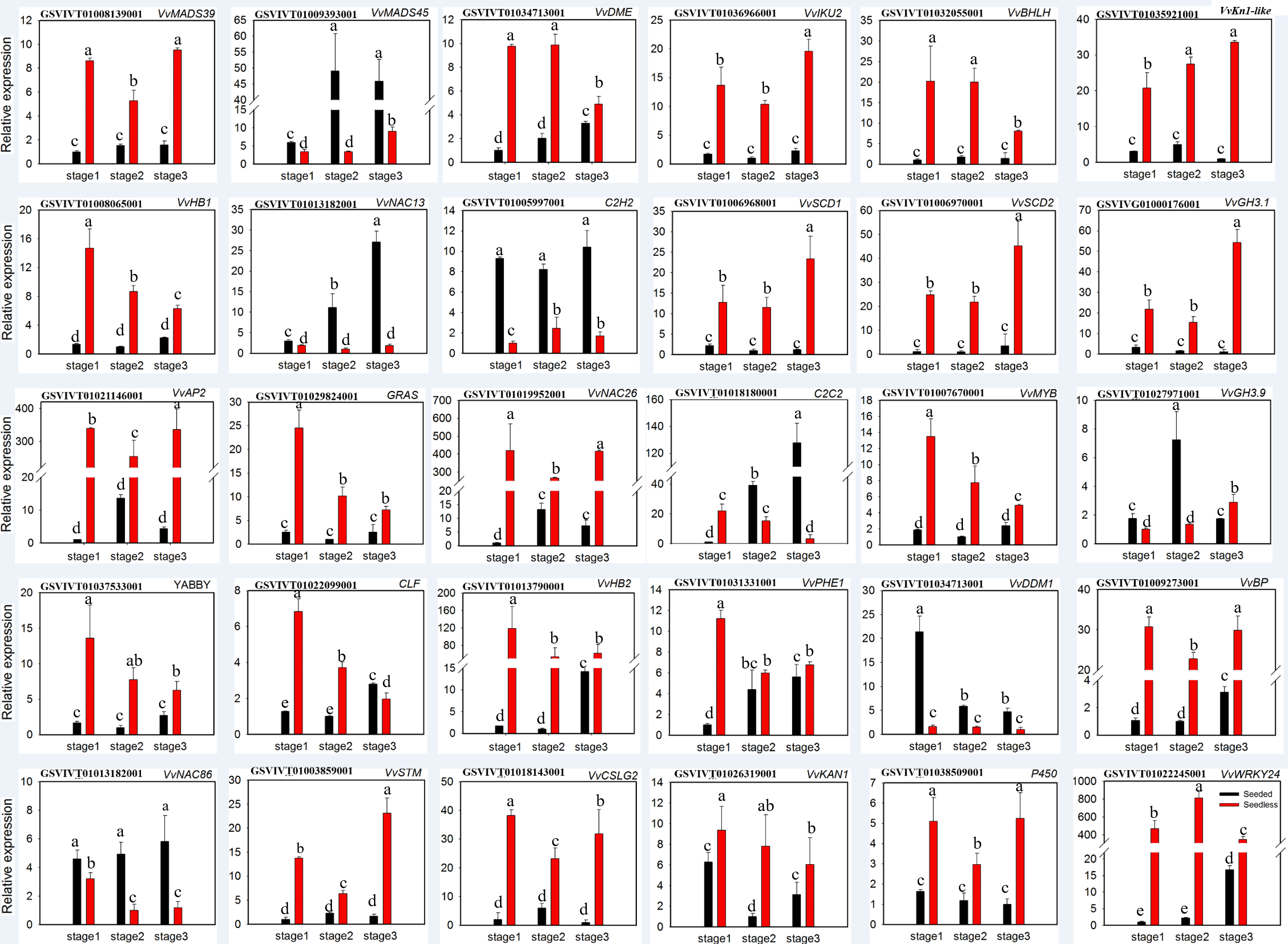

Supplement: Additional file 9: Figure S4. — Verification of RNA-Seq results by q RT-PCR. Thirty genes were selected for validation of the RNA-Seq data by qRT-PCR. ‘Seeded’ represents ‘seeded progeny used for RNA-Seq’, ‘Seedless’ represents ‘seedless progeny used for RNA-Seq’. (PNG 962 kb) [file 12864_2016_3193_MOESM9_ESM.png]

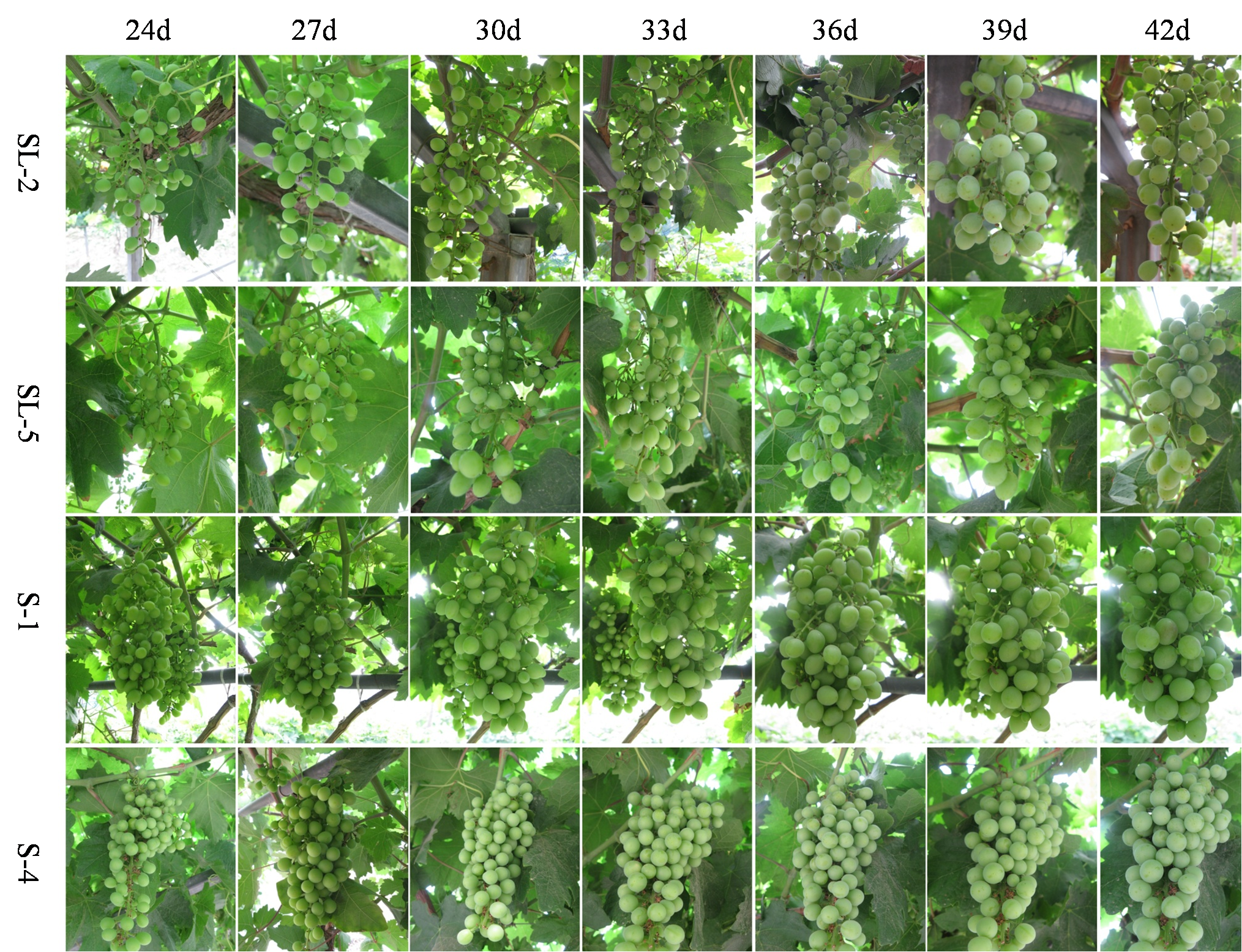

Supplement: Additional file 11: Figure S5. — Clusters of seeded and seedless grape progeny during sample collecting. ‘S’ represents ‘seeded’ and ‘SL’ ‘seedless’. Representative images are shown for each stage. (PNG 8508 kb) [file 12864_2016_3193_MOESM11_ESM.png]
